# Genome-Wide Identification and Preliminary Functional Analysis of *BAM* (β-Amylase) Gene Family in Upland Cotton

**DOI:** 10.3390/genes14112077

**Published:** 2023-11-14

**Authors:** Yanlong Yang, Fenglei Sun, Penglong Wang, Mayila Yusuyin, Wumaierjiang Kuerban, Chengxia Lai, Chunping Li, Jun Ma, Fei Xiao

**Affiliations:** 1 Research Institute of Economic Crops, Xinjiang Academy of Agricultural Sciences, Urumqi 830091, China; yangyl0629@163.com (Y.Y.); mayila324@163.com (M.Y.); lchxia2001@163.com (C.L.); chunpin96@126.com (C.L.); 2 Xinjiang Academy of Agricultural Sciences Kuqa County Upland Cotton Test Station, Xinjiang Academy of Agricultural Sciences, Kuqa 842000, China; 17591619607@163.com (P.W.); 13999661527@126.com (W.K.); 3 State Key Laboratory of Cotton Biology, Institute of Cotton Research of the Chinese Academy of Agricultural Sciences, Anyang 455000, China; xjsunfenglei@163.com; 4 Xinjiang Key Laboratory of Biological Resources and Genetic Engineering, College of Life Science and Technology, Xinjiang University, Urumqi 830046, China

**Keywords:** β-amylase (*BAM*) gene family, upland cotton, genome-wide identification, fiber development, starch metabolism

## Abstract

The β-amylase (*BAM*) gene family encodes important enzymes that catalyze the conversion of starch to maltose in various biological processes of plants and play essential roles in regulating the growth and development of multiple plants. So far, *BAMs* have been extensively studied in *Arabidopsis thaliana* (*A. thaliana*). However, the characteristics of the *BAM* gene family in the crucial economic crop, cotton, have not been reported. In this study, 27 *GhBAM* genes in the genome of *Gossypium hirsutum L* (*G. hirsutum*) were identified by genome-wide identification, and they were divided into three groups according to sequence similarity and phylogenetic relationship. The gene structure, chromosome distribution, and collinearity of all *GhBAM* genes identified in the genome of *G. hirsutum* were analyzed. Further sequence alignment of the core domain of glucosyl hydrolase showed that all *GhBAM* family genes had the glycosyl hydrolase family 14 domain. We identified the *BAM* gene *GhBAM7* and preliminarily investigated its function by transcriptional sequencing analysis, qRT-PCR, and subcellular localization. These results suggested that the *GhBAM7* gene may influence fiber strength during fiber development. This systematic analysis provides new insight into the transcriptional characteristics of *BAM* genes in *G. hirsutum*. It may lay the foundation for further study of the function of these genes.

## 1. Introduction

Starch is the most crucial form of carbohydrate storage in plants, and plant growth and development depend on starch metabolism [1]. Starch is temporarily stored in chloroplasts, seeds, and other specialized starch storage organs for a long time [2,3]. Starch is degraded by a series of enzymes (including α-amylase (*AMY*), β-amylase (*BAM*), limit dextrinase *(PUL*), β-glucosidase and α-glucan phosphorylase (*PHO*)) to release primary chemical energy and organic matter for plant growth, development, and response to abiotic stress [4,5,6,7,8,9]. The β-amylase (*BAM*) family is named for the ability to catalyze the hydrolysis of starch into maltose units by its catalytic members. It is the main starch-degrading enzyme in plant tissues [10,11]. β-Amylases (*BAMs*) are essential enzymes that catalyze the conversion of starch into maltose and play important roles in regulating plant growth, development, and abiotic stress tolerance [12]. They are vital enzymes for transient starch degradation in chloroplasts and important molecules for gene regulation [13,14,15]. β-Amylase (*BAM*) is an exoamylase that catalyzes the hydrolysis of α-1,4-linked oligosaccharides and polyglucans. It is mainly responsible for the hydrolysis of stored starch and the degradation of transitional starch, resulting in β-limit dextrin and β-maltose [11]. However, this family also contains proteins with weak catalytic activity, additional domains, or no localization with starch substrates. β-Maltose is exported from chloroplasts at night and is the main product of starch decomposition in leaves [16]. *BAM*s are also the only enzymes that can produce β-maltose in plants. *BAM* is a glycosyl hydrolase 14 family member with a conserved glycosyl hydrolase 14 domain. It is widely distributed in various plants and some microorganisms and is encoded by a multi-gene family [17,18,19].

*BAM* exists as a gene family in many plants. To date, genome-wide analysis has identified nine members of the *BAM* gene family (*GFMs*) in *A. thaliana* [8,20], 10 in rice varieties [21], 13 in maize varieties, 11 in *Brachypodium distachyon* varieties, 10 in sorghum varieties, 10 in foxtail millet varieties [22], 16 in banana varieties [11], 10 in potato varieties, 8 in trifoliate orange varieties [23], and 17 in pear varieties [9]. Most studies on the *BAM* family’s functions are carried out in *A. thaliana*. Maltose is the main product of starch degradation [24]. In *Arabidopsis*, BAMs are the main hydrolases for starch decomposition at night, which act on the non-reducing end of the α-1,4-linked glucan chain to produce maltose [10]. In *Arabidopsis*, nine genes encode *BAM*-like proteins, more than any other starch-metabolizing enzyme, and other plant genomes contain a similar number of *BAM* genes [25]. The analysis of conserved intron sites of *BAM* genes in terrestrial plants revealed that the family contains two subfamilies, one subfamily contains *BAM*1/3/9, and the other subfamily contains *BAM*2/4/8 [26]. So far, their characterization in *Arabidopsis* has revealed an alarming degree of sub-functionalization and neo-functionalization [27]. However, during the evolution of vascular plants, the *BAM* gene family has undergone diversity, resulting in isomers with different spacer structures and biological activities [15].

Cotton is one of the critical economic crops in the world and plays a vital role in China’s economy. Cotton fiber is the leading financial component of cotton, and fiber quality is an essential factor determining the economic quality of cotton [28]. Cotton fiber is a single-cell structure formed by the differentiation and development of the outer epidermis of cotton ovules. It is also an ideal single-cell model for cell elongation and cell wall modification. In the process of cotton fiber development, 15 days post-anthesis (DPA) is a crucial period, mainly for the fiber elongation stage, followed by the secondary wall thickening stage [29,30]. The BAM gene family plays a crucial role in transient starch metabolism [3], seed germination [31], and growth and development [32], and the development of cotton fiber is accompanied by the synthesis and degradation of starch, so BAM genes may play important regulatory roles. The role of the BAM gene family in fiber development is unclear, despite its vital importance. In this study, 27 *GhBAM* genes were identified in the whole genome of upland cotton. The *GhBAM* gene family was analyzed for its protein physicochemical properties, chromosome location, gene structure, conserved motifs, domain alignment, and phylogenetic evolution. In addition, the functions and evolutionary characteristics of the *GhBAM* gene family were explored by promoter element analysis and expression analysis of *GhBAM* family genes. The results provide a reference for future studies of the structure and function of the *GhBAM* gene family, as well as the identification and characterization of the *BAM* gene family in other species, and also provide a theoretical basis for further study of the molecular mechanism of fiber development in upland cotton.

## 2. Materials and Methods

### 2.1. Identification and Phylogenetic Analysis of the GhBAM Gene Family in G. hirsutum

The amino acid sequences of the *Arabidopsis BAM* gene family were downloaded from the TAIR website (https://www.Arabidopsis.org/; accessed on 30 September 2023), and the *Arabidopsis BAM* protein sequence was used as an index to perform local sequence alignment in the upland cotton genome (TM-1_V2.1, ZJU) [33]. The obtained candidate sequences were submitted to the NCBI-CDD website (https://www.ncbi.Nlm.Nih.gov/cdd; accessed on 30 September 2023) and the HMMER website (https://www.ebi.ac.uk/Tools/hmmer/search/hmmsearch; accessed on 30 September 2023) to verify whether they contained the Glyco_hydro_14 (PF01373) conserved domain. In Ex PASy (https://web.expasy.org/compute_pi; accessed on 30 September 2023), the average number of amino acids, molecular weight, isoelectric point, and hydrophilicity were obtained. The online tool WoLF PSORT (https://wolfpsort.hgc.jp/; accessed on 30 September 2023) was used to predict the subcellular localization of proteins encoded by *GhBAM* gene family members.

### 2.2. Chromosome Distribution, Synteny, Ka/Ks, and Phylogenetic Analysis of the GhBAM Family

The location information of the gene on the chromosome was obtained from the GFF3 data in the genome, and the TBtools tool was used to draw the schematic diagram and analyze gene collinearity. The non-synonymous substitution (Ka) and synonymous substitution (Ks) rates of the *BAM* gene family were calculated to analyze the selection pressure in the evolutionary process. All sequences were aligned using the default settings of Clustal X2.1, and phylogenetic trees were constructed using the MEGA-7 (http://www.megasoftware.net/mega-7; accessed on 30 September 2023) neighbor-joining (NJ) method, one thousand bootstrap samplings were performed, and other parameters were default values.

### 2.3. Analysis of GhBAM Gene Domain and Conserved Motif

The *BAM* gene structure of upland cotton was analyzed online using the GSDS website (http://gsds.Cbi.Pku.edu.cn; accessed on 30 September 2023). The MEME online tool (http://meme-suite.org/tools/meme; accessed on 30 September 2023) was used to identify the conserved motifs of the *GhBAM* gene family, setting 6–50 motif widths and up to 12 motifs. TBtools software (V 1.068) was used for visual analysis of gene structure and conserved motifs.

### 2.4. Subcellular Localization and Promoter Element Analysis of GhBAM7

The *GhBAM7-GFP* vector was constructed. The tobacco was cultured in a greenhouse for three–four weeks, and activated *Agrobacterium* containing the target gene subcellular targeting vector was added to resistant LB liquid medium (kanamycin 50 mg/L, rifampicin 25 mg/L). The OD value was measured between 0.8 and 1.0, and the bacterial liquid was centrifuged and resuspended. The bacterial liquid was re-suspended at room temperature for one–three hours to prepare for infection. The third and fourth leaves from the top were selected for infection (infection between the two veins). The plants, after injection, were cultured in the dark for 24 h and then cultured normally for two days. The GFP fluorescence near the injection site of the leaves was observed by fluorescence microscopy. The sequence of about 2000 bp upstream of the start codon of the *GhBAM* family genes was determined as a regulatory promoter region database. These sequences were then uploaded to the PlantCARE website (http://bioinformatics.psb.ugent.be; accessed on 30 September 2023) for the identification and analysis of cis elements.

### 2.5. GhBAM Gene Expression Analysis

Transcriptome data for ovular and fibrous tissue were also downloaded from the NCBI Sequence Read Archive collection PRJNA490626. The cotton introgression line population was planted in Shihezi City, Xinjiang Uygur Autonomous Region. After flowering, fiber samples at 15 DPA, 20 DPA, and 25 DPA of extreme individuals (Xinluzhong 60 (strong fiber strength), Xinhai 36 (weak fiber strength), and extreme materials for hybrid progeny) were immediately frozen in liquid nitrogen and stored in an ultra-low temperature refrigerator at −80 °C. The samples were sent to the gene sequencing company. Based on the Illumina Hi Seq sequencing platform, RNA-seq technology was used to identify the differentially expressed genes of fiber development in the two introgression lines. Real-time quantitative (RT-qPCR) preliminary verification was performed. The expression abundance (fragments per kilobase per million, FPKM) value of *GhBAM* was obtained from the transcriptome data. The log_2_ (FPKM + 1) formula calculated the degree of expression difference, and the heat map of gene expression was drawn using the Heat Map program in TBtools software (V 1.068).

## 3. Results

### 3.1. Identification and Sequence Retrieval of BAM Gene Family in Upland Cotton

In order to identify the *BAM* genes of upland cotton (*G. hirsutum*), BLASTP was performed using the glycosyl hydrolase family 14 domain of the reported *A. thaliana* sequence. BLAST comparison of the glycosyl hydrolase family 14 domains resulted in 29 sequences. When the BLAST results were further analyzed, only 27 genes contained the expected glycosyl hydrolase family 14 domain and were used for further analysis. The glycosyl hydrolase family 14 domain in these proteins was located at the N-terminal. Sequences of glycosyl hydrolase family 14 domains and isotypes of the same genes were excluded.

The Pfam database further confirmed the presence of the glycosyl hydrolase family 14 domain in 27 selected genes. The Pfam entry number of the glycosyl hydrolase family 14 domain is PF01373.2. The Pfam results showed that the glycosyl hydrolase family 14 domain comprised 402 amino acids. NCBI CDD analysis further confirmed the existence of the glycosyl hydrolase family 14 domain and showed that all *GhBAM* genes contained the glycosyl hydrolase family 14 domain. These genes were renamed *GhBAM1*–*GhBAM27* (Table S1) according to their genomic location on the A and D subgenome chromosomes.

The relevant information of the *GhBAM* genes is shown in Table S1, including gene id, gene location, direction, isoelectric point (PI), molecular weight (Mw), subcellular location, peptide, genome, and coding sequence (CDS) sequence length (Table S1). Subcellular localization analysis showed that *GhBAM* family members were mainly located in the nucleus, cytoplasm, and chloroplast. Moreover, there were significant differences in protein length, molecular weight (MW), and isoelectric point (pI). *BAM* protein in upland cotton had an average length of 497 amino acids and ranged in length from 72 (*GhBAM19*) to 703 (*GhBAM16*) (Table S1). The isoelectric points (pIs) and molecular weights of the *BAM* protein sequences in upland cotton were 4.39–9.59 and 7904.94–79,049.31 Da, respectively. The overall mean of all *BAM* protein hydrophilic (GRAVY) scores was negative, indicating that the *BAM* protein was hydrophilic (Table S1).

### 3.2. BAM Family Sequence Comparison and Phylogenetic Tree Analysis

**Figure 1:**
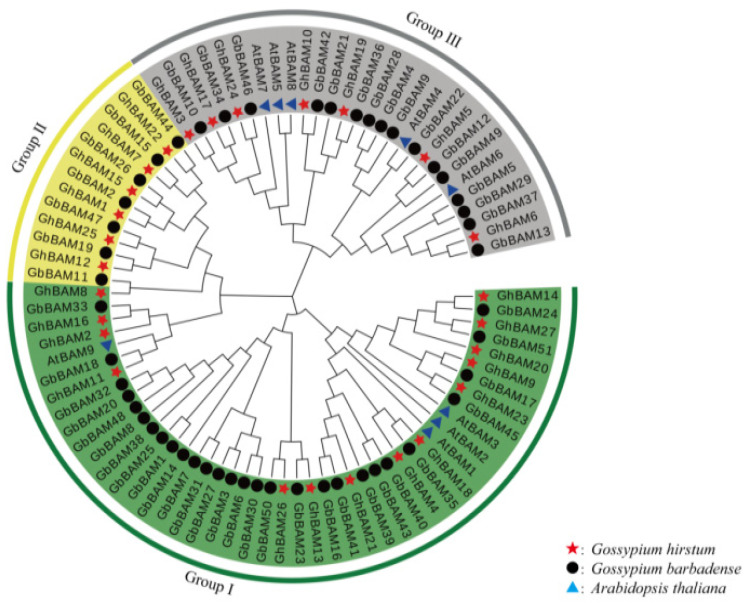
Evolutionary analysis of *BAM* gene family in upland cotton, Sea Island cotton, and *A. thaliana*.

Conserved sequence alignment was used for phylogenetic analysis. This included 27 *BAM* sequences of upland cotton, 9 of *A. thaliana*, and 51 of Sea Island cotton (*Gossypium barbadense*). The phylogenetic tree showed that 87 *BAM* genes were classified into three categories: I, II, and III. The results are shown in Figure 1. Group I had the most significant number of *BAM* genes, including 14 members in upland cotton, 28 in Sea Island cotton, and 4 members in *Arabidopsis*. Group II had 13 members, mainly including six sequences of upland cotton and seven sequences of Sea Island cotton. Group III had 7 sequences of upland cotton, 16 sequences of Sea Island cotton, and 5 sequences of *A. thaliana* (Figure 1).

### 3.3. BAM Gene Structure and Conserved Motif Prediction

**Figure 2:**
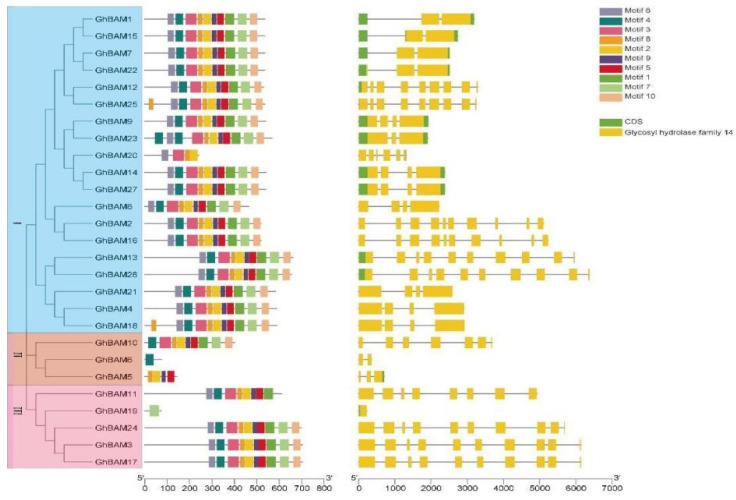
Analysis of motif and gene structure of *BAM* gene family in upland cotton.

Exon and intron predictions can help to further understand the structural evolution of the *GhBAM* gene family. Genome and CDS sequences were compared to obtain the structures of exons and introns. The results showed that all genes in Group I had more than three exons interrupted by four introns. *GhBAM5* and *GhBAM6* in Group II contained three and two exons, respectively, interrupted by four and three introns. In Group I, the intron length of *GhBAM26* and *GhBAM13* was greater than that of the remaining *GhBAM* genes. The number and length of introns and exons of *GhBAM3*, *GhBAM17,* and *GhBAM24* in Group III were similar, but the length of introns was different. Among all *GhBAM* gene family members, Group III had longer *GhBAM* introns (Figure 2).

An online MEME web server was used to analyze the conserved motifs in the *GhBAM* protein family. The results showed that the more similar the type and number of patterns within a group, the more tightly they functioned. Still, there were some differences between members of different groups. Some patterns were mainly present in different groups (Figure 2). Motifs 1, 2, 3, 5, 6, 8, 9, and 10 were conserved in all *GhBAM* proteins, with motifs 1, 2, 3, 5, 6, 8, 9, and 10 missing only in *GhBAM5* and *GhBAM19*. Members of Group I (*GhBAM20*) contained motifs 6, 3, 8, and 2, while *GhBAM18* and *GhBAM25*, also belonging to Group I, contained motif 8, suggesting that some functions changed during evolution. The members of Group II (*GhBAM5*, *GhBAM6*, and *GhBAM10*) were composed of motifs 4, 3, 8, 2, 9, 5, 1, 7, and 10. Motif 6 was missing from Group II members. Among members of Group III (*GhBAM3*, *GhBAM11*, *GhBAM17*, *GhBAM19*, and *GhBAM24*), motifs 6, 4, 3, 8, 2, 9, 5, and 1 appeared in *GhBAM11*. The consistent sequence of motifs retrieved from the MEME suite (Table S2) showed that of all ten identified motifs, motif 4 and motif 7 were associated with the glycosyl hydrolase family 14 domain (Figure 2). Motifs 4 and 7 were present in all *GhBAM* proteins, further confirming that the glycosyl hydrolase family 14 domain was present in all proteins.

### 3.4. Prediction of Cis-Acting Elements in the GhBAM Gene Promoter Region

**Figure 3:**
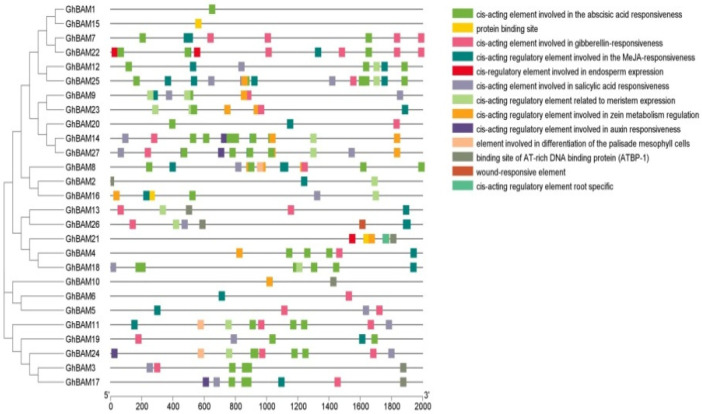
Analysis of promoter cis-acting elements of *BAM* gene family in upland cotton.

Cis-acting element analysis is critical to understanding gene function and regulation because cis-acting elements in the promoter region regulate gene expression. Various cis-regulatory elements were found in the 2000 bp promoter region upstream of the start codon (ATG). In the *GhBAM* gene family, differences existed in the 14 cis-acting elements of the promoter (Figure 3). The functional dependencies of the elements involved in these cis-elements included protein-binding sites, hormones, cell differentiation, and elements involved in plant development and growth (Figure 3). There were 16 cis-elements at the binding site of DNA-binding protein (ATBP-1), 3 root-specific cis-elements, 803 hormone-responsive cis-elements (gibberellin, salicylic acid, abscisic acid, MeJA, and auxin), and 5 cis-elements involved in stress (trauma). There were 153 cis-elements involved in plant growth and development (related to meristem expression, protein-binding sites, regulation of zein metabolism, endosperm expression, regulation of specific seeds, and differentiation of palisade mesophyll cells) (Figure 3).

### 3.5. Chromosome Localization, Gene Replication, Collinearity Analysis, and Selection Pressure Analysis

**Figure 4:**
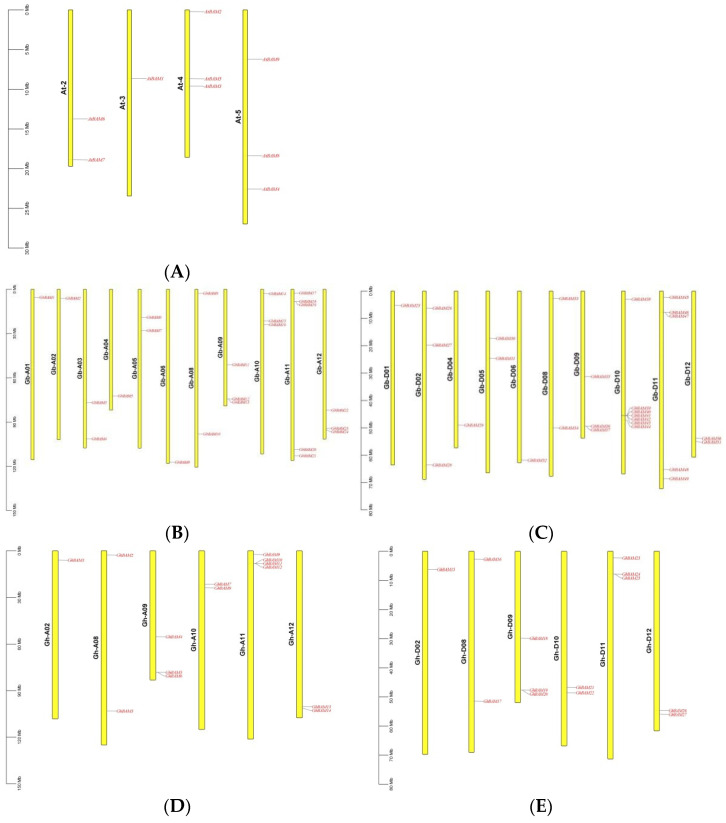
Chromosome localization analysis of *BAM* gene family. (**A**) *A. thaliana BAM* gene mapping, (**B**) Sea Island cotton At subgenomic *BAM* gene mapping, (**C**) Sea Island cotton Dt subgenomic *BAM* gene mapping (**D**) Upland cotton At subgenomic *BAM* gene mapping, (**E**) Upland cotton Dt subgenomic *BAM* gene mapping.

A genome-wide analysis of *G. hirsutum* to determine the location of the *GhBAM* gene on the chromosome revealed that *GhBAM* genes were unevenly distributed in the A and D subgenomes (Figure 4). Genes were named according to their order in the A and D subgenomes. Fourteen of these genes (*GhBAM1*, *GhBAM2*, *GhBAM3*, *GhBAM4*, *GhBAM5*, *GhBAM6*, *GhBAM7*, *GhBAM8*, *GhBAM9*, *GhBAM10*, *GhBAM11*, *GhBAM12*, *GhBAM13*, *GhBAM14*) were located in the A subgenomic chromosome, and 13 genes (*GhBAM15*, *GhBAM16*, *GhBAM17*, *GhBAM18*, *GhBAM19*, *GhBAM20*, *GhBAM21*, *GhBAM22*, *GhBAM23*, *GhBAM24*, *GhBAM25*, *GhBAM26*, *GhBAM27*) were located in the D subgenomic chromosome above (Figure 4). *GhBAM1* was mapped to A02; *GhBAM2* and *GhBAM3* were mapped to A08; *GhBAM4*, *GhBAM5*, and *GhBAM6* were mapped to A09; *GhBAM7* and *GhBAM8* were mapped to A10; *GhBAM9*-*12* was mapped to A11; and so on. *GhBAM13* and *GhBAM14* were mapped to A12 (Figure 4). On the other hand, *GhBAM15* was on D02; *GhBAM16* and *GhBAM17* were on D08; *GhBAM18*, *GhBAM19,* and *GhBAM20* were on D09; *GhBAM21* and *GhBAM22* were on D10; *GhBAM23*, *GhBAM24*, and *GhBAM25* were on D11; and so on. *GhBAM26* and *GhBAM27* were on D12 (Figure 4). On some At and Dt subgenomic chromosomes, the deletion of the *GhBAM* gene may have been due to gene loss during evolution.

**Figure 5:**
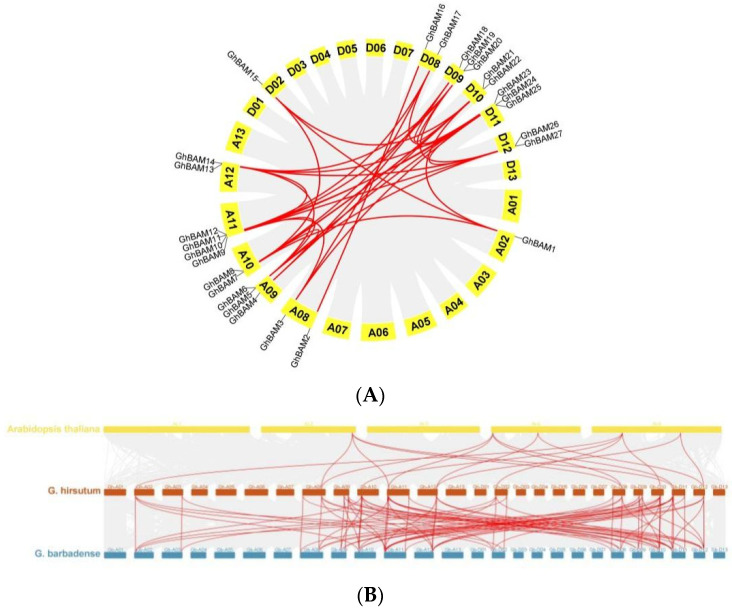
Collinearity analysis of *BAM* gene family. (**A**) *BAM* gene family analysis in upland cotton; (**B**) Collinearity analysis among *Arabidopsis thaliana*, upland cotton, and Sea Island cotton (collinear blocks of other plant genomes in the gray background). In contrast, the red line indicates the gene pairs of the *BAM* gene.

In plant evolution, replication, including tandem and segmentary replication, is the main force driving gene expansion. The amplification mechanism of *BAM* genes in *G. hirsutum* was determined through gene replication analysis, as shown in Figure 5 and Table S3. All genes were segmented among the 34 para-homologous gene pairs. These results indicated that tandem replication was essential in *GhBAM* gene amplification. In order to analyze the collinearity of the *BAM* genes in cotton, the MCScanX technique was used to detect the collinearity of upland cotton with tetraploid cotton varieties (*G. barbadense* and *A. thaliana*). Collinearity analysis revealed that *G. hirsutum* and *A. thaliana* had 16 homologous gene pairs (Figure 5B), and *G. hirsutum* and *G. barbadense* had 86 homologous gene pairs (Figure 5B).

To estimate the correlation of repeating genes over a long evolutionary history, Ka/Ks values in *GhBAM* gene homologous pairs were calculated based on different selection pressures such as purification, neutral, and active selection. According to the Ka/Ks analysis, the Ka/Ks values were below 1.0, indicating that these *GhBAM* genes underwent strong purification selection during evolution (Table S3). When differentiation is limited by purification selection, gene replication pairs may perform similar functions.

### 3.6. Transcriptome Analysis

#### 3.6.1. Analysis of *GhBAM* Gene Expression in Different Tissues of Upland Cotton

**Figure 6:**
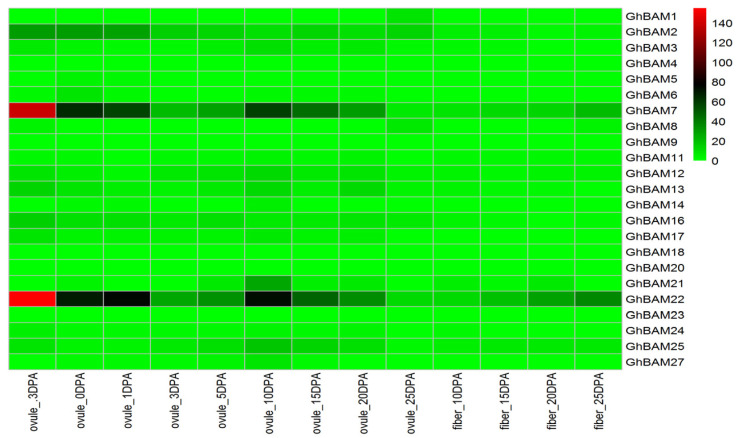
Transcriptional expression analysis of *BAM* gene family in ovule and fiber tissues of upland cotton.

Tissue-specific expression analysis of *GhBAM* genes in ovule and fiber tissues showed that the expression of these genes in different tissues was different (Figure 6). *GhBAM2*, *GhBAM7*, and *GhBAM22* were mainly expressed in the ovule and fiber, with higher levels at −3 d, 0 d, 1 d, 10 d, 15 d, and 20 d in the ovule (Figure 6). In addition, *GhBAM2*, *GhBAM7*, and *GhBAM22* were expressed at 10 d, 15 d, and 25 d during fiber development. The remaining genes were poorly expressed in tissues at these three stages of ovule and fiber development (Figure 6).

#### 3.6.2. Transcriptional Expression Analysis of *GhBAM* Genes at 15 and 20 Days of Fiber Development in Extreme Materials

**Figure 7:**
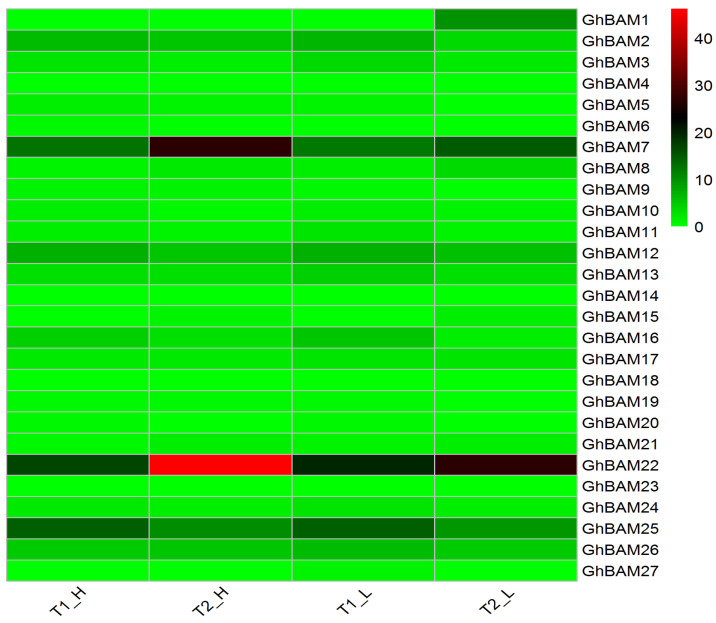
Transcriptional expression analysis of extreme materials with different fibers at day 15 and day 20 (T1_H: 15 days Xinluzhong 60 fiber; T2_H: 20 days Xinluzhong 60 fiber; T1_L: 15 days Xinhai 20 fiber; T2_L: 20 days Xinhai 20 fiber).

Transcriptional expression analysis of the *GhBAM* genes at day 15 and day 20 of fiber development in extreme materials showed that some genes were highly expressed (Figure 7), indicating that the *GhBAM* genes may play roles in fiber development periods. *GhBAM1* had high expression at 20 d in extreme materials with low fiber length (Figure 7). The expression levels of *GhBAM7* and *GhBAM22* in extreme materials with high fiber length at 15 d and 20 d were higher than those in extreme materials with low fiber length. The expression difference at 20 d was highly significant (Figure 7). In contrast, the expression of *GhBAM25* at the 15 d stage was higher than that at the 20 d stage in extreme materials. The expression of *GHBAM25* in extreme materials with high fiber length was higher than in extreme materials with low fiber length (Figure 7).

### 3.7. qRT-PCR Validation of the GhBAM Gene Family in Two Extreme Materials

**Figure 8:**
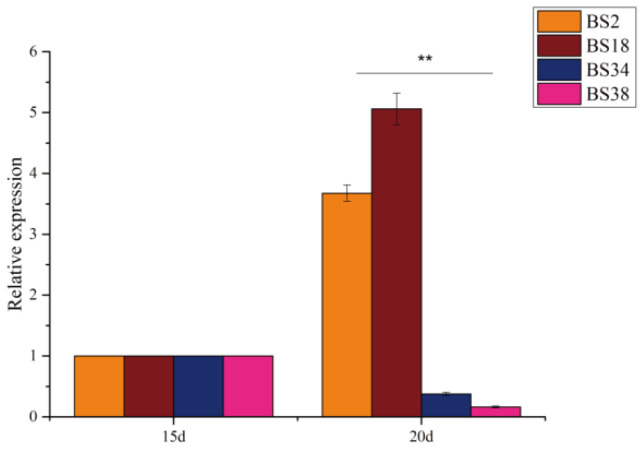
qRT-PCR analysis of *GhBAM7* gene from four extreme materials at 20 days (20 d) of fiber development (BS2, BS18, BS34 and BS38: extreme materials for hybrid progeny). ** are significantly different at the 0.05 level of significance.

Information about gene function can be provided by analysis of gene expression levels. To analyze the roles of the *GhBAM* genes in fiber development, from the results obtained from the RNA-seq data analyzed in the previous section (Figure 6 and Figure 7), the *GhBAM7* gene was selected because it may be involved in fiber growth and development. The expression levels of the *GhBAM7* gene at 15 and 20 days of fiber development in four extreme materials were analyzed by qRT-PCR. The gene-specific primers are shown in Table S4. The results of qRT-PCR analysis showed that the expression patterns of the *GhBAM7* gene in these four extreme materials changed at day 15 and day 20 (Figure 8). BS2 and BS18 showed high expression at 20 d, while BS24 and BS38 showed little change at 20 d. The results showed that the *GhBAM7* gene was induced at 20 d of fiber development and reached the peak expression level quickly (Figure 8). These results suggest that the *GhBAM7* gene may be involved in fiber development at 20 d.

### 3.8. Subcellular Localization Analysis of GhBAM7 Protein

**Figure 9:**
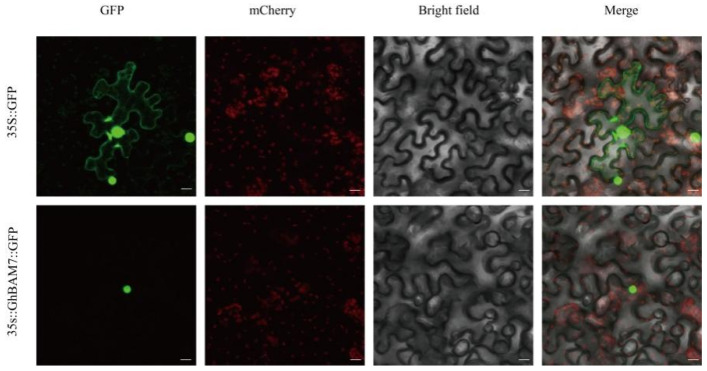
Subcellular localization analysis of *GhBAM7*.

Combined with the differential expression pattern and tissue specificity of the *BAM* gene family in cotton fiber development, we selected the *GhBAM7* gene that was highly expressed at day 15 and day 20 during fiber development for in-depth study. In order to determine the location of the *GhBAM7* protein in cells, a GFP vector for *GhBAM7* protein subcellular localization analysis was constructed. At the same time, empty GFP fusion protein was used as the control, and the position of the fusion protein was observed by fluorescence confocal microscopy three days after injecting tobacco leaves. The results showed that *GHBAM7-GFP* was distributed in the nucleus and that the *GhBAM7* protein was located in the nucleus (Figure 9).

## 4. Discussion

As an essential source of natural fiber, cotton is of great significance to the study of fiber quality and yield [34]. Completing cotton genome sequencing enables us to further study the fiber yield and quality mechanisms. The conversion of starch and sugar plays a vital role in fiber formation. Biochemical and genetic analyses have shown that the functions of *BAM* genes are related to plant germination, growth, development, and maturation. Studies in *Arabidopsis* show that β-amylase (*BAM*, EC3.2.1) is the main starch-degrading enzyme [11]. Therefore, studying the function and related roles of the *GhBAM* gene family is very important.

In conclusion, except for in-depth research on the *BAM* gene family of *Arabidopsis* in model plants, research on other plants is relatively shallow. In this study, based on the whole genome sequencing data of upland cotton, members of the *GhBAM* gene family were identified and screened, and their structure and function were analyzed in depth, providing a theoretical basis for further research on the role of the *GhBAM* gene family in the growth and development of cotton. So far, 9 *BAM* genes in *Arabidopsis* have been identified, including 10 in rice (*Oryza sativa* L.), 13 in maize (*Zea mays* L.), 11 in Brachypodium distachyon (*Brachypodium distachyon* (L.) *P. Beauv*), 10 in sorghum (*Sorghum bicolor* (L.) *Moench*), 10 in millet (*Setaria italica* var. *germanica (Mill.) Schred*), 16 in banana (*Musa nana Lour.*), 10 in potato (*Solanum tuberosum* L.), and 8 in trifoliate (*Citrus trifoliata* L.) [11,21,22,35,36]. Therefore, this study systematically analyzed the *BAM* gene family of upland cotton. *BAM* genes were distributed on 21 chromosomes in upland cotton and on 12 chromosomes in Sea Island cotton, among which several *GhBAM* genes were densely distributed on chromosomes with high accumulation, suggesting that tandem duplication and chromosome segment duplication may have contributed to the expansion of *BAM* gene family. The results of collinearity analysis show that 34 *GhBAM* genes showed collinearity in upland cotton, followed by 16 *GhBAM* genes showing collinearity with Arabidopsis *BAM* genes. It is speculated that *BAM* family genes may be involved in various growth and development processes regulated by hormone-related response elements. In order to determine the expression characteristics of the *GhBAM7* protein in cells, the gene was analyzed by subcellular localization, and the GFP vector for *GhBAM7* protein subcellular localization analysis was constructed. *GhBAM7-GFP* fusion protein was injected into tobacco leaves, and the position of the fusion protein was observed by fluorescence confocal microscopy three days after injection, with unloaded GFP as the control. The results showed (Figure 9) that *GHBAM7-GFP* was distributed in the nucleus, indicating that the *GhBAM7* protein was located in the nucleus.

Arabidopsis *AtBAM4* has been shown to participate in the starch degradation process [37]. Transcriptomic data analysis showed that *GhBAM7* and *GhBAM22* were more expressed at 15 d and 20 d in fiber than other genes. Moreover, the expression levels at 15 d and 20 d in extreme materials with high fiber lengths were higher than in extreme materials with short fiber lengths. The *GHBAM7* gene was selected, and the expression levels at 15 and 20 days of fiber development in the four extreme materials were analyzed by qRT-PCR. The results of qRT-PCR analysis showed (Figure 8) that the expression patterns of the *GhBAM7* gene in these four extreme materials changed at day 15 and day 20. BS2 and BS18 showed high expression at 20 d, while BS24 and BS38 showed little change at 20 d. The results showed that the *GhBAM7* gene was induced at 20 d of fiber development and reached the peak expression level quickly (Figure 8). These results suggested that the *GhBAM7* gene may be involved in fiber development at 20 days. It is speculated that it may play a role in starch degradation during the development of upland cotton fiber.

This study identified 27 *GhBAM* genes and analyzed their phylogenetic relationships, gene structures, protein motifs, and expression patterns at different stages of cotton fiber development. This comprehensive study adds to our understanding of how *BAM* genes are involved in the development process of upland cotton fibers and will provide an essential basis for future research using *BAM* for crop improvement.

## 5. Conclusions

*BAM* family genes were identified in four cotton cultivars, and their evolutionary relationships were analyzed with a phylogenetic tree. The gene structure, phylogenetic relationship, cis-acting elements, and collinearity of *GhBAMs* in upland cotton were analyzed, which increases the understanding of the *BAM* gene family in upland cotton. The analysis of cis-acting elements suggested that *BAM* genes might be involved in plant growth, development, glucose metabolism, and hormone signal transduction. Tissue-specific analysis of all *GhBAM* family genes combined with transcriptome analysis revealed that two genes were specifically expressed in extreme materials, and these two materials were also highly expressed in extreme materials with long fiber lengths. Four progeny extreme materials were selected for qRT-PCR verification, and the results showed that expression was high in the long progeny extreme materials and low in the poor extreme materials (with a very significant difference between them). It was concluded that the *GhBAM7* gene might be involved in the development of upland cotton fiber, which provides a theoretical basis for studying the molecular mechanisms of *BAM* genes in upland cotton fiber development.

## Data Availability

Data are contained within the article.

## Supplementary Materials

The following supporting information can be downloaded at: https://www.mdpi.com/article/10.3390/genes14112077/s1, Table S1: Characteristics of BAM gene family in G. hirsutum; Table S2. Multilevel consensus sequences of motifs in GhBAM; Table S3 Analysis of Ka, Ks and Ka/Ks of BAM gene family in upland cotton; Table S4 Primers used in GhBAM7 gene experiments.
